# Imaging of the saccule for the diagnosis of endolymphatic hydrops in Meniere disease, using a three-dimensional T2-weighted steady state free precession sequence: accurate, fast, and without contrast material intravenous injection

**DOI:** 10.1186/s41747-017-0020-7

**Published:** 2017-10-09

**Authors:** Aïna Venkatasamy, Francis Veillon, Aude Fleury, Michael Eliezer, Maher Abu Eid, Benoit Romain, Hella Vuong, Dominique Rohmer, Anne Charpiot, Henri Sick, Sophie Riehm

**Affiliations:** 1 Service d’imagerie 1, Hôpitaux Universitaires de Strasbourg, 1 avenue Molière, Strasbourg, F-67098 France; 2 Service d’ORL, Hôpitaux Universitaires de Strasbourg, Strasbourg, France; 3 EA3430, Strasbourg University, FMTS, 3 Avenue Moliere, 67000 Strasbourg, France; 4 Institut d’Anatomie Normale, Hôpitaux Universitaires de Strasbourg, Strasbourg, France

**Keywords:** Saccule, Endolymphatic hydrops, Magnetic resonance imaging (MRI), Normal anatomy, Meniere disease

## Abstract

**Background:**

Endolymphatic hydrops can be studied on magnetic resonance imaging (MRI) using images acquired 4 h after intravenous injection of Gd-chelate. Our aim was to compare high-resolution T2-weighted images of the saccule in normal subjects with histological sections from cadavers and to identify its changes in Meniere disease, compared to healthy volunteers.

**Methods:**

Sixty-four healthy volunteers without any otologic disease and 64 patients who fulfilled all the criteria for unilateral Meniere disease underwent 3 T MRI using a T2-weighted steady state free precession (SSFP) sequence, without contrast material injection. Images of healthy volunteers were compared with histological sections of normal inner ears from premature foetuses and compared with volunteers.

**Results:**

The normal saccule was easily visible on T2-weighted images in volunteers, with a normal maximal height of 1.6 mm (1.4 ± 0.1 mm, mean ± standard deviation) and a good correlation with reference histological sections, while in Meniere disease the saccule was dilated in 52/62 patients (84%), with a saccular height greater than 1.6 mm (1.69 ± 0.24 mm, *p* = 0.001), found in 45/52 patients (86%). An associated increased width (greater than 1.4 mm) was found in 23/52 patients (44%). A round shape or the non-visualisation of the saccule were also found in 2/52 (4%) and in 5/62 patients (8%), respectively.

**Conclusions:**

A T2-weighted sequence is an easy method to diagnose Meniere disease. Saccular abnormalities were found in 84% of the cases: elongation (height > 1.6 mm) in 86%, increased saccular width in 44%, or a missing saccule in 8%.

## Key points

- A normal saccule is always visible on T2-weighted SSFP sequence at 3 T
- A coronal section through the anterior and external ampullas is the most useful image
- Eighty-four percent (52/62) of patients with Meniere disease presented with saccular abnormalities
- Three useful signs: saccular height > 1.6 mm, increased saccular width, or missing saccule

## Background

**Fig. 1:**
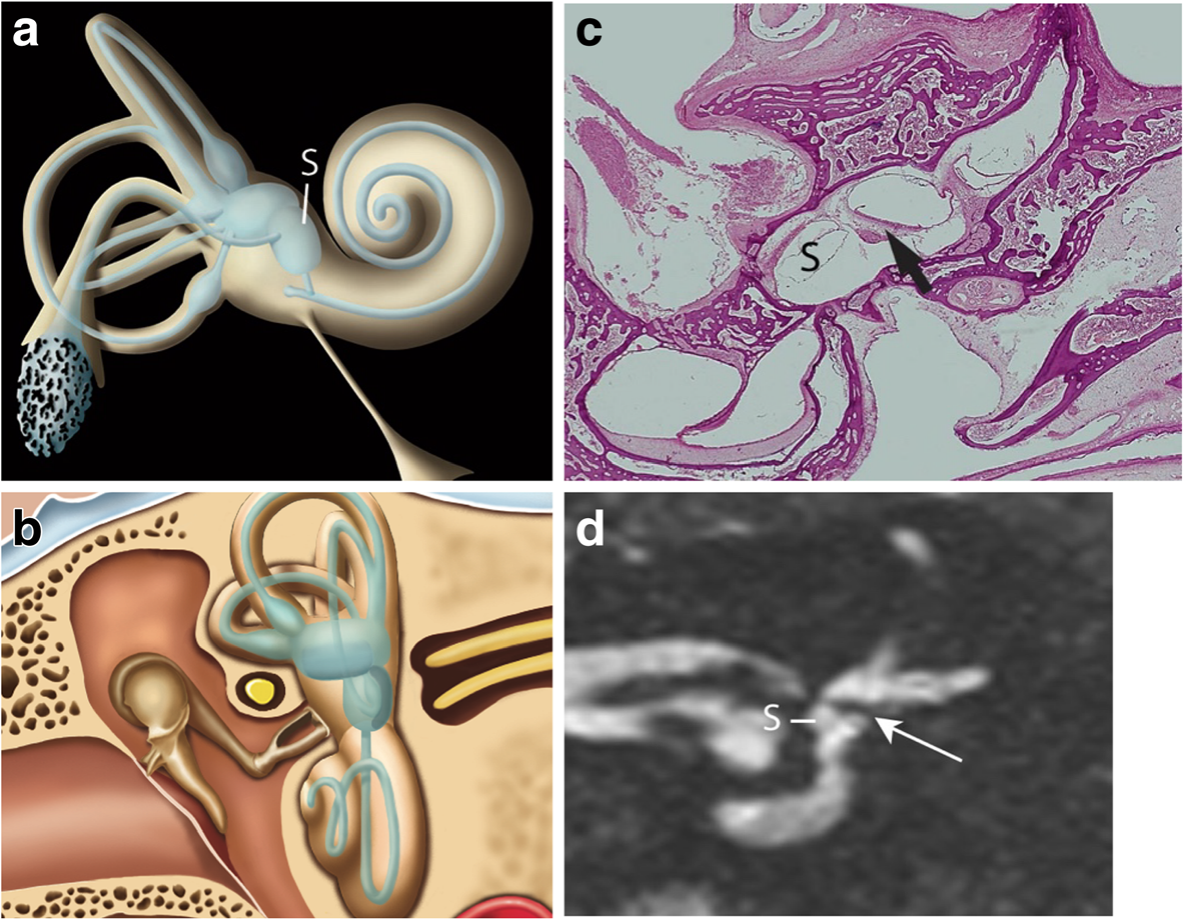
**a** Oblique lateral drawing of the membranous and bony labyrinth showing the saccule (S). **b** Schematic coronal drawing of the membranous and bony labyrinth. **c** Coronal histological section through the anterior part of the vestibule showing the saccule (S) below the utricular macula (arrow). **d** 3 T MRI. Coronal T2-weighted gradient-echo FIESTA-C image through the anterior part of the vestibule showing the saccule (S) and the hypointense signal of the utricular macula (arrow), as described in the equivalent histological section shown in **c**

Meniere disease is a chronic disease of unknown aetiology, causing vertigo, hearing loss, sensation of clogged ear, and tinnitus. The diagnosis is based on a combination of the patient’s symptoms, the results of the clinical examination, and functional tests [1]. Knowledge of normal anatomy of the saccule is important in order to identify size and shape modifications in pathological conditions. Meniere disease symptoms are known to be correlated to the degree of endolymphatic hydrops, which is the distension of endolymph-filled structures (Fig. 1a) [2, 3]. The endolymphatic hydrops can be easily studied with 3 T magnetic resonance imaging (MRI). However, all published studies used delayed images acquired 4 h after intravenous injection of a Gd-chelate or 24 h after intratympanic injection of a Gd-chelate [4–10].

Our aim was to compare high-resolution T2-weighted images of the saccule in normal subjects with histological sections from cadavers and to identify its changes in Meniere disease compared to normal subjects.

## Methods

The Ethics Committee of our institution approved the study (Internal Research Project, authorisation number HUS-PRI 5012). Patients and healthy volunteers gave their written informed consent to participate in the study. The temporal bones of premature foetuses born after 7 months of gestation were provided by the Anatomical Institute of the Strasbourg University.

### Study population

Patients who presented at our institution with all criteria for unilateral definite Meniere disease according to the 2015 classification provided by the American Academy of Otolaryngology—Head and Neck Surgery (AAO-HNS) [1] were prospectively enrolled from 01/01/2014 to 01/01/2016. Patients were evaluated by auditory and vestibular functional specific tests for Meniere disease [2]. Exclusion criteria were atypical Meniere disease (which did not meet all criteria of the AAO-HNS classification), associated otological pathologies and the impossibility to perform the MRI examination [1, 2].

In addition, 64 healthy volunteers without any past or present otologic symptoms were enrolled for comparison with the histological sections and with the patient group.

### MRI protocol

All subjects underwent an axial high-resolution T2-weighted three-dimensional gradient-echo steady state free precession (SSFP) sequence, specifically a fast imaging employing steady-state acquisition (FIESTA-C) sequence at 3 T (Signa HDxt, General Electric, Strasbourg, France), using an 8-channel head coil. This sequence is a modified SSFP sequence, which does not require any contrast injection.

Patients and volunteers were asked not to swallow during the image acquisition in order to avoid motion artefacts. The study box was placed parallel to the orbital roof, thus enabling all images to be acquired directly in the plane of the lateral semi-circular canal on axial images. The acquisition parameters were as follows: repetition time 7 ms; echo time 2.8–1.2 ms; field of view 220 × 198 mm; frequency × phase 484 × 484; flip angle 60°; number of excitations 1, bandwidth 83.3 kHz; isotropic voxel size 0.3 × 0.3 × 0.3 mm. The acquisition time was 7 min and 49 s.

### Histological sections

Histological sections of normal inner ears were obtained from temporal bones of five premature foetuses born after 7 months of gestation, originating from the Anatomical Institute of the University of Strasbourg. Notably, at this gestation stage, the development of the inner ear cavities and ossicles has reached its normal adult size and aspect [11]. The temporal bone was removed *en bloc*. One foetus was sectioned on a coronal plane, one on a axial plane, and three on a sagittal plane. This material was cut into histological slices, from 0 to 12 μm in thickness, and slices were stained with haematoxylin-eosin and each of the 250 slices was examined under a magnifying glass. We reviewed all histological sections to choose the anatomical plane of reference for our MRI slices. The chosen reference plane for the coronal sections was perpendicular to an axial plane (parallel to the orbital roof) and went from the apex of the petrous bone to the posterior part of the mastoid (Fig. 1b, c).

### Image analysis

**Fig. 2:**
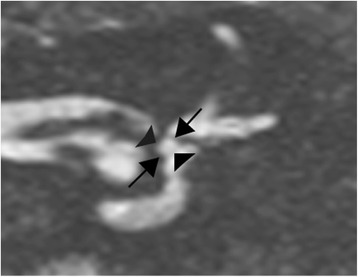
3T MRI. Coronal T2-weighted gradient-echo FIESTA-C image, through the anterior and external ampullas. Example of the measurements of the saccular height (arrows) and width (arrowheads)
- the saccule was measured from the middle of the lateral wall to the inner part of the medial wall of the adjacent vestibule, as shown in Fig. 2.

Coronal and sagittal reconstructions were obtained from the original axial dataset of the three-dimensional T2-weighted sequence. All measurements were carried out using the open-source OsiriX software (available at http://www.osirix-viewer.com) [12]. Two radiologists specialised in head and neck imaging (5 years and 35 years of experience) read the T2-weighted images, blinded to the clinical presentation (Meniere disease patients and volunteers were presented in a random order to the reader by the investigator). In particular:

- the saccule was analysed on a coronal section through the anterior part of the anterior vestibule as shown in Fig. 1b, d;
- the height of the saccule was measured along its axis, from the inferior part of the utricular macula down to the lower part of the saccule, as shown in Fig. 2;

Both sides were analysed in Meniere disease patients and in the control group.

### Statistical analysis

Continuous variables were expressed as mean and standard deviation (SD). Categorical variables were expressed in terms of numbers and percentages. The interobserver agreement for the visual analysis was calculated using Cohen’s κ for categorical variables and intraclass correlation coefficient (ICC) for quantitative variables. The intraobserver agreement was calculated using ICC for quantitative variables and categorical variables. A Student *t*-test was performed to compare the means between the patients with Meniere disease and the healthy volunteers. A Fisher exact test was used to test the correlation between the presence of an endolymphatic hydrops and the time of completion of the MRI after the last crisis. The significance was set at *p* = 0.050. All tests were performed using the Statistical Package for the Social Sciences, SPSS software (version 22.0 IBM Inc., Armonk, New York, USA).

## Results

The clinical features of the patients and healthy volunteers are given in Table 1.

**Table 1:** Clinical characteristics of Meniere disease patients and healthy volunteers

	Meniere disease group	Control group
Patients included in the study	64	64
Patients excluded due to motion artefacts	2	4
Number of analysed patients	62	60
Sex ratio	25 men	30 men
	37 women	30 women
Mean age (range)	51 years (13–82)	32.3 years (22–57)
Average duration of the disease	37 months	Not applicable
	(15 days–17 years)	
Average length between MRI and the last crisis	5.7 months	Not applicable
MRI performed < 3 months after the last crisis	43 patients (69.3%)	Not applicable
MRI performed between 3 and 6 months after the last crisis	12 patients (19.3%)	Not applicable
MRI performed > 6 months after the last crisis	7 patients (11.3%)	Not applicable

On the histological sections, the saccule occupied the inferior, medial, and anterior part of the vestibule, facing the oval window, located under the utricle and medially to the perilymph. It had the oval shape of a rugby ball, lying in a coronal plane through the anterior vestibule at the level of the lateral and anterior ampullas, as shown in Fig. 2. It followed the craniocaudal axis of the vestibule and its orientation was close to that of the anterior semicircular canal. Its medial wall was made of the saccular macula, which was not visible, as it blended in the osseous adjacent vestibule. On the histological sections in a coronal plane, the measurements were 1.6 mm for the saccular height and 0.9 mm for the saccular width.

The main results between the Meniere disease group and the control group composed of healthy volunteers and the histological analysis of the saccule are given in Table 2.

**Table 2:** Comparison among Meniere disease patients, control group, and histological sections at the same section level

	Meniere disease patients (n = 62)	Control group (n = 60)	Histological sections
Visibility of the saccule	Visible in 57 (92%) Not visible in 5 (8%)	Visible in 60 (100%)	Visible in 100% Reference section
Radiological hydrops	Present in 52 (84%)	Not applicable	Not applicable
Height Height mean ± SD (range)	Increased in 45 (86%) 1.68 ± 0.24 mm (1.20–2.30)	ᅟ 1.4 mm ± 0.10 (1.10–1.60 mm)	1.6 mm
Width ᅟ Width mean ± SD (range)	Increased in 23 (44%) Isolated increase in 2 (4%) 1.30 ± 0.21 mm (1.10–1.70)	Not applicable ᅟ 1.20 ± 0.13 mm (0.9–1.40 mm)	Not applicable ᅟ 0.9 mm
Bilateral involvement	Present in 19 (35%)	Not applicable	Not applicable

**Fig. 3:**
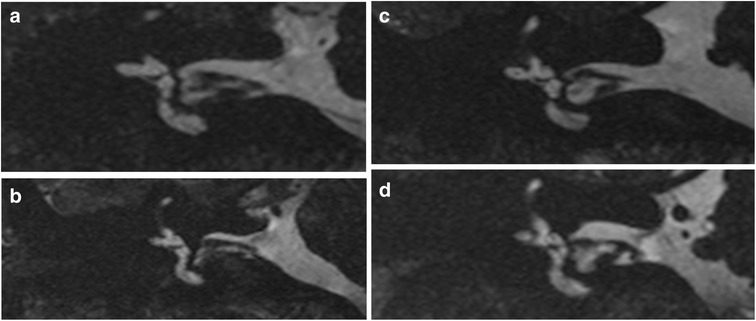
3T MRI. Four different healthy volunteers from the control group: **a**, **b**, **c** and **d**. Coronal T2-weighted gradient-echo FIESTA-C images through the anterior and external ampullas, showing a normal saccule (white arrows)

The images of 60 healthy volunteers were analysed as four of them were excluded due to motion artefacts. In these healthy subjects, the saccule was always visible (100% of the cases) on the reference coronal section with excellent interobserver correlation (κ = 1.0). It always presented with the ovoid shape of a rugby ball, as observed on the equivalent histological sections, occupying the anterior and medial quadrant of the vestibule (under the linear hypointense signal of the utricular macula), as shown in Fig. 3. Its lateral wall was distant from the footplate, separated by the perilymphatic cistern and its craniocaudal axis appeared to be nearly in the same direction as the anterior semicircular canal. The vertical saccular macula, located close to the medial wall of the bony vestibule, was not visible on MRI, as it blended with the hypointense signal of the surrounding bony vestibule on the T2-weighted images. In volunteers, the mean height of the saccule was 1.40 ± 0.10 mm (mean ± SD) and the maximal height was 1.6 mm, with a range from 1.10 to 1.60 mm (Fig. 5); the mean normal width of the saccule was 1.20 ± 0.10 mm and the maximal width was 1.40 mm. The height measurements in volunteers were similar for both observers, with an ICC of 0.89 on the right side and 0.68 on the left side; so were the width measurements, with an ICC of 0.86 on the right side and 0.72 on the left side. The intraobserver agreement on volunteers was also good with an ICC of 0.87 for the height measurement and 0.77 for the width.

**Fig. 4:**
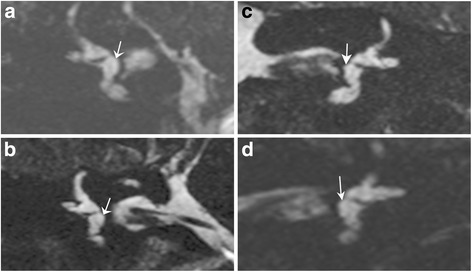
3T MRI. Four different patients suffering from Meniere disease: **a**, **b**, **c** and **d**. Coronal T2-weighted gradient-echo FIESTA-C images through the anterior and external ampullas, showing four dilated saccules with an increased saccular height > 1.6 mm (white arrows)

**Fig. 5:**
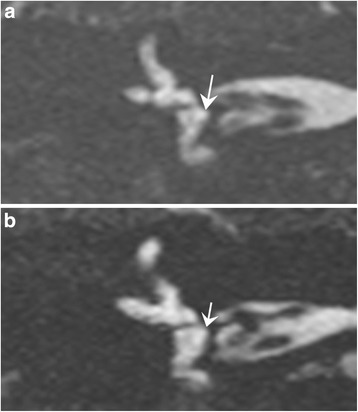
3T MRI. Coronal T2-weighted gradient-echo FIESTA-C images in two patients suffering from Meniere disease. 5 **a**) Dilated saccule with an increased saccular height (> 1.6 mm), associated with an increased saccular width (> 1.4 mm). 5 **b**) Round saccule with an isolated increased saccular width > 1.4 mm

Sixty-two patients with Meniere disease were analysed as 2 patients were excluded because of motion artefacts (mostly related to swallowing). Fifty-two of the 62 patients with Meniere disease (84%) presented with signs of saccular hydrops. The measurement on the coronal image was carried out easily, with an average of 2 min and 30 s for each patient. The saccular height was increased (greater than 1.6 mm) in 45 out of 52 cases (86%) and gave the saccule an elongated oval shape (Fig. 4). The mean saccular height in Meniere disease was 1.69 mm ± 0.24 (mean ± SD) with a range from 1.20 to 2.30 mm, which was significantly different from the control group (1.40 ± 0.10 mm, range from 1.10 to 1.60 mm, *p* < 0.001) or the measures on the histological sections (1.60 mm) (Figs. 2, 3, 4 and 5). The interobserver agreement for the evaluation of the hydrops in Meniere disease patients was good (ICC 0.69 on the right side and 0.84 on the left) and the intraobserver agreement was good with an ICC of 0.87.

The saccular width was also increased (greater than 1.40 mm) in 23 of 52 cases (44%) (Fig. 5a, b). The width of the saccule in the Meniere group was 1.30 ± 0.21 mm (range 1.10–1.70 mm), which was significantly different from the control group (1.20 ± 0.13 mm, range 0.90–1.40 mm, *p* < 0.001) or the histological sections (0.9 mm). A round shape appearance of the saccule with an isolated increase of the saccular width (4% of the cases, n = 2) was also considered as pathological (Fig. 5b), as the normal saccule always presented with an oval shape in the control group. The interobserver agreement was very good (ICC 0.72 for the right side and 0.94 for the left side). The intraobserver agreement was good, with an ICC of 0.86.

Bilateral saccular involvement was found in 19 of 52 cases (35%) and the vast majority had no clinical signs on the contralateral side. Furthermore, in cases of bilateral involvement; the clinically affected side showed no greater distension compared to the contralateral side.

**Fig. 6:**
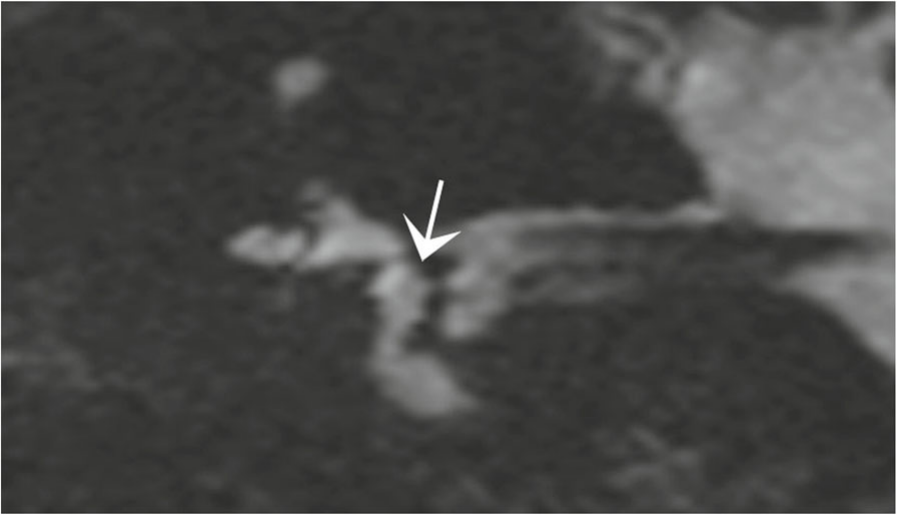
3T MRI. Coronal T2-weighted gradient-echo FIESTA-C in a patient suffering from Meniere disease. The saccule is not clearly visible (arrow), although there are not visible motion artefacts. The absence of visualisation of the saccule can be considered as pathological, as the saccule was always seen in the controls

In 8% of the cases (n = 5), the saccule on the side of the clinical symptoms was poorly defined (Fig. 6), while the contralateral saccule was clearly visible, without any artefact interfering with its interpretation. As the saccule was visible on both sides in the control group, a non-visualisation of the saccule on the clinical pathological side, observed only in Meniere disease patients, was considered as pathological.

The presence of an endolymphatic hydrops on MRI was correlated:

- to the audiogram and vestibular tests (Table 3)

**Table 3:** Correlation between the endolymphatic saccular hydrops to audiogram and vestibular tests results in Meniere disease patients

	Saccular hydrops on MRI (n = 52)	No saccular hydrops on MRI (n = 10)
Normal hearing	18 (35%)	4 (40%)
Hearing loss	34 (65%)	6 (60%)
Mild to moderate (20–70 dB)	34 (65%)	5 (50%)
Severe (> 70 dB)	0 (0%)	1 (10%)
Normal vestibular test	15 (29%)	2 (20%)
Not performed	2 (4%)	1 (10%)
Unilateral hypovalence on vestibular test	35 (67%)	7 (70%)
- to the time of completion of the MRI after the last Meniere crisis (Table 4)

**Table 4:** Correlation between the endolymphatic hydrops and time of completion of the MRI after the last crisis in 62 Meniere patients

	Endolymphatic hydrops	No endolymphatic hydrops
MRI < 3 months after the last crisis (n = 43)	35 (81%)	8 (19%)
MRI > 3 months after the last crisis (n = 19)	16 (84%)	3 (16%)

Fisher exact test did not find a correlation between the presence of a hydrops and the time of completion of the MR examination
- to the duration of the disease (Table 5)

**Table 5:** Correlation between the endolymphatic hydrops and the disease duration in 62 Meniere patients

	Endolymphatic hydrops	No endolymphatic hydrops
Disease duration < 2 years (n = 43)	39 (91%)	4 (9%)
Disease duration > 2 years (n = 19)	13 (68%)	6 (32%)

Fisher exact test did not show any significant difference between the presence of the hydrops and the duration of the disease

The Fisher exact test did not find a correlation between the degree of saccular dilation and the hearing loss, the vestibular dysfunction, duration of the disease, and the delay of completion of the MRI.

## Discussion

The essential point of our method is the fast measurement of the saccule, enabling us to visualise a saccular hydrops in 84% of patients with Meniere disease, in agreement with previously published data [4–13]. In Meniere disease, the saccule appeared elongated with an increased average height, round shape, or in few cases not visualised on the side of the clinical symptoms, compared to normally shaped and sized saccules in the control group and on histological sections, where it was always visible and oval-shaped. The non-visualisation of the saccule in Meniere disease has a possible histopathological explanation. In fact, Kimura and Schucknecht [14] and Schucknecht and Rüther [15] described ruptures and fistulas of the membranous labyrinth on temporal bone sessions, which could cause this non-visualisation.

The endolymphatic hydrops associated with Meniere disease was firstly described by Hallpike and Cairns in 1938 [16] and confirmed by Altman and Fowler in 1965 [17]. In a more recent study on temporal bones, Rauch et al. [18] described the involvement of the saccule in almost all cases of endolymphatic hydrops. According to Horner [19], the degree of saccular dilation appeared correlated to the degree of membranous distortion towards the footplate. This was confirmed by Morita et al. [20], who described a saccular hydrops on the pathological side in Meniere disease patients, while the cochlear and utricular hydrops were less frequent.

To our knowledge, our study is the first one to analyse both size and morphology of the saccule in patients with Meniere disease without intravenous or intratympanic contrast material injection. Most of the authors used fluid attenuated inversion recovery (FLAIR) or three-dimensional inversion-recovery turbo spin-echo sequences, acquired 4 h after intravenous Gd-based contrast material injection [5, 6, 20–24]. Naganawa et al. [8] have been able to individualise the endolymph from the perilymph using a three-dimensional inversion-recovery turbo spin-echo sequence after intravenous injection of a Gd-based contrast material, but they were unable to separate the utricle from the saccule, contrary to Attye et al. [5]. One of the downsides of these methods using contrast injection is the time needed for the penetration of the Gd-based contrast material in the perilymph (approximately 4 h) while the required injection also exposes the patient to potential Gd-related risks. The main advantage of the method proposed by Naganawa et al. [8] is given by the morphological visualisation of the anterior membranous labyrinth, although Attye et al. [5] gave examples of dilation of the cochlear duct in their normal population; thus, the significance of a dilated cochlear duct is uncertain. On the contrary, in our study the saccule only appeared dilated in Meniere disease and no pathological dilation of the saccule was observed in the control group.

Other authors, such as Seo et al. [25], Sun et al. [9], or Fiorino et al. [10], used FLAIR images acquired 24 h after intratympanic gadolinium injection. Seo et al. [25], using MRI images acquired 24 h after intratympanic injection, found a hydrops in 81% of their patients (21/26) with saccular involvement in the majority of the cases (19 patients, 69%).

Our method enables a fast diagnosis of Meniere disease in less than 8 minutes. Its other major strength is that it does not require intravenous or intratympanic injection of a Gd-based contrast material. The major downside of the T2-weighted gradient-echo SSFP sequence (FIESTA-C) may be its sensitivity to motion artefacts. In our study, it involved 6/128 subjects (5%), mostly related to swallowing. When compared to the literature, all sequences used for the imaging of the membranous labyrinth are quite long: 9 minutes in Attye et al. [5] and 14 min in Sun et al. [9], compared with 7 min and 49 s in our study.

Notably, three-dimensional T2-weighted turbo-spin-echo sequences (such as SPACE, CUBE, VISTA) would also work for the measurement of the saccule as long as the special resolution is sub-millimetric, but unlike gradient-echo sequences, spin-echo sequences are not sensitive to fluid composition [26, 27]. Thus, using gradient-echo SSFP sequences, such as FIESTA-C in our study, true fast imaging with steady state precession (trueFISP) sequences, or constructive interference steady state (CISS), sequences, additional information can be obtained, such as signal changes of the liquids of the inner ear that might be seen in case of schwannomas or meningiomas of the internal auditory canal, or labyrinthitis [26, 27].

To conclude, a simple 8-minute T2-weighted gradient-echo SSFP sequence (FIESTA-C), performed at 3 T, is a fast and anatomically accurate method to diagnose Meniere disease without injection of Gd-based contrast material. The measures are sub-millimetric, but the interobserver and intraobserver agreements were good, with κ ranging from 0.68 to 0.94. In Meniere disease, an endolymphatic hydrops with saccular abnormality was present in 84% of the cases, an elongated saccule (height > 1.60 mm) in 86% of the cases, an increased saccular width (>1.40 mm) in 44% of the cases (isolated in 4%), a missing saccule in 8% of the cases.

## Abbreviations

FIESTA: Fast imaging employing steady-state acquisition
ICC: Intraclass correlation coefficient
MRI: Magnetic resonance imaging
SPSS: Statistical package for the social sciences

## References

[1] Goebel JA (2016). Equilibrium Committee Amendment to the 1995 AAO-HNS Guidelines for the Definition of Meniere’s Disease. Otolaryngol Head Neck Surg.

[2] Lopez-Escamez JA, Carey J, Chung WH (2015). Diagnostic criteria for Meniere’s disease. J Vestib Res.

[3] Salt AN, Plontke SK (2010). Endolymphatic hydrops: pathophysiology and experimental models. Otolaryngol Clin N Am.

[4] Pender DJ (2014). Endolymphatic hydrops and Menière’s disease: a lesion meta-analysis. J Laryngol Otol.

[5] Attye A, Eliezer M, Boudiaf N et al (2017) MRI of endolymphatic hydrops in patients with Meniere’s disease: a case-controlled study with a simplified classification based on saccular morphology. Eur Radiol 27:3138–3146 10.1007/s00330-016-4701-z 27999985

[6] Gürkov R, Pyykö I, Zou J, Kentala E (2016). What is Menière's disease? A contemporary re-evaluation of endolymphatic hydrops. J Neurol.

[7] Naganawa S, Satake H, Kawamura M (2008). Space visualization of endolymphatic space, perilymphatic space and bone by a single pulse sequence; 3D-inversion recovery imaging utilizing real reconstruction after intratympanic Gd-DTPA administration at 3 Tesla. Eur Radiol.

[8] Naganawa S, Komada T, Fukatsu H, Ishigaki T, Takizawa O (2006). Observation of contrast enhancement in the cochlear fluid space of healthy subjects using a 3D-FLAIR sequence at 3 Tesla. Eur Radiol.

[9] Sun W, Guo P, Ren T, Wang W (2017). Magnetic resonance imaging of intratympanic gadolinium helps differentiate vestibular migraine from Ménière disease. Laryngoscope 127:2382–2388 10.1002/lary.26518 28220492

[10] Fiorino F, Pizzini FB, Beltramello A, Barbieri F (2011). MRI performed after intratympanic gadolinium administration in patients with Ménière's disease: correlation with symptoms and signs. Eur Arch Otorhinolaryngol.

[11] Anson B, Donaldson J (1981). Surgical anatomy of the temporal bone.

[12] Kim G, Jung H-J, Lee H-J (2012). Accuracy and reliability of length measurements on three-dimensional computed tomography using open-source OsiriX Software. J Digit Imaging.

[13] Lin MY, Timmer FCA, Oriel BS (2006). Vestibular evoked myogenic potentials (VEMP) can detect asymptomatic saccular hydrops. Laryngoscope.

[14] Kimura RS, Schuknecht HF (1975). Effect of fistulae on endolymphatic hydrops. Ann Otol Rhinol Laryngol.

[15] Schuknecht HF, Rüther A (1991). Blockage of longitudinal flow in endolymphatic hydrops. Eur Arch Otorhinolaryngol.

[16] Hallpike C, Cairns H (1938). Observations on the pathology of Meniere’s syndrome. J Laryngol Otol.

[17] Altman F, Fowler E (1943). Histological findings of Meniere’s syndrome complex. Ann Otol Rhinol Laryngol.

[18] Rauch SD, Merchant SN, Thedinger BA (1989). Meniere’s syndrome and endolymphatic hydrops. Double-blind temporal bone study. Ann Otol Rhinol Laryngol.

[19] Horner KC (1993). Review: morphological changes associated with endolymphatic hydrops. Scanning Microsc.

[20] Morita N, Kariya S, Farajzadeh Deroee A (2009). Membranous labyrinth volumes in normal ears and Ménière disease: a three-dimensional reconstruction study. Laryngoscope.

[21] Naganawa S, Sugiura M, Kawamura M (2008). Imaging of endolymphatic and perilymphatic fluid at 3 T after intratympanic administration of gadolinium-diethylene-triamine pentaacetic acid. Am J Neuroradiol.

[22] Nakashima T, Naganawa S, Sugiura M (2007). Visualization of endolymphatic hydrops in patients with meniere’s disease. Laryngoscope.

[23] Naganawa S, Nakashima T (2014). Visualization of endolymphatic hydrops with MR imaging in patients with Meniere’s disease and related pathologies: current status of its methods and clinical significance. Jpn J Radiol.

[24] Naganawa S, Yamazaki M, Kawai H (2010). Visualization of endolymphatic hydrops in Meniere’s disease with single-dose intravenous gadolinium-based contrast media using heavily T2-weighted 3D-FLAIR. Magn Reson Med Sci.

[25] Seo YJ, Kim J, Choi JY, Lee WS (2013). Visualization of endolymphatic hydrops and correlation with audio-vestibular functional testing in patients with definite Meniere’s disease. Auris Nasus Larynx.

[26] Ishikawa K, Haneda J, Okamoto K (2013). Decreased vestibular signal intensity on 3D FIESTA in vestibular schwannomas differentiating from meningiomas. Neuroradiology.

[27] Venkatasamy A, Le Foll D, Karol A (2017). Differentiation of vestibular schwannomas from meningiomas of the internal auditory canal using perilymphatic signal evaluation on T2-weighted gradient-echo fast imaging employing steady state acquisition at 3 T. Eur Radiol Exp.

